# Multimodal Imaging in Ophthalmology

**DOI:** 10.1155/2016/3120129

**Published:** 2016-08-10

**Authors:** Lisa Toto, Roberto dell'Omo, Gian Marco Tosi, Giuseppe Querques, Sandrine A. Zweifel, Antoine Labbé

**Affiliations:** ^1^Ophthalmology Clinic, University “G. d'Annunzio” of Chieti-Pescara, 66100 Chieti, Italy; ^2^University of Molise, 86100 Campobasso, Italy; ^3^Ophthalmology Section, Department of Medicine, Surgery and Neuroscience, University of Siena, 53100 Siena, Italy; ^4^Department of Ophthalmology, University Paris Est Creteil, Centre Hospitalier Intercommunal de Creteil, 94010 Creteil, France; ^5^Department of Ophthalmology, University Vita-Salute, IRCCS Ospedale San Raffaele, Via Olgettina, 60 20132 Milan, Italy; ^6^Department of Ophthalmology, University Hospital Zurich, University of Zurich, Zurich, Switzerland; ^7^Department of Ophthalmology III, Quinze-Vingts National Ophthalmology Hospital, 75012 Paris, France

In recent years the use of different, established, and novel imaging techniques provided detailed insight into several retinal diseases. These modalities provide information about anatomy and functional changes in the retina with high resolution images which improve diagnosis and management of retinal pathologies.

The special issue on multimodal imaging in ophthalmology published seven clinical research articles.

Among published articles R. R. Bastos et al. evaluated the usefulness of multimodal image analysis to characterize vitelliform lesions in adult-onset foveomacular vitelliform dystrophy and acquired vitelliform patients. R. Mastropasqua et al. used intraoperative and postoperative spectral domain optical coherence tomography (SD-OCT) to evaluate anatomical results after two different ocriplasmin injection procedures for the treatment of vitreomacular traction. P.-M. Lêvèque et al. used OCT angiography to detect changes in optic nerve head vascularization in glaucoma patients. E.V. Boiko and D. S. Maltsev compared retromode scanning laser ophthalmoscopy images and OCT central retinal thickness maps as a guide for macular laser photocoagulation in patients with macular edema. L. M. Brandao et al. compared two different spectral-domain OCT systems in regard to full macular thickness and ganglion cell layer-inner plexiform layer measures and in regard to structure-function correlation when compared to standard automated perimetry. Q. Liang et al. evaluated the relationship between corneal and conjunctival epithelium thickness evaluated with SD-OCT and ocular surface clinical tests in dry eye disease patients.

I. Yuvaci et al. assessed the effects of mydriatics on choroidal thickness evaluated by means of SD-OCT with Enhanced Depth Imaging modality and anterior chamber changes evaluated using a Pentacam Scheimpflug camera system. All manuscripts underwent a peer review process before publication.

*Lisa Toto* *Lisa Toto* *Roberto dell'Omo* *Roberto dell'Omo* *Gian Marco Tosi* *Gian Marco Tosi* *Giuseppe Querques* *Giuseppe Querques* *Sandrine A. Zweifel* *Sandrine A. Zweifel* *Antoine Labbé* *Antoine Labbé*

